# From Olive Oil Lovers to Mediterranean Diet Lifestyle Followers: Consumption Pattern Segmentation in the Portuguese Context

**DOI:** 10.3390/nu16234235

**Published:** 2024-12-07

**Authors:** Valentina Chkoniya, Maria João Gregório, Sandra Filipe, Pedro Graça

**Affiliations:** 1 GOVCOPP, ISCA-UA, University of Aveiro, 3810-193 Aveiro, Portugal; 2 Civil Engineering Department, ISISE, University of Coimbra, 3030-788 Coimbra, Portugal; 3 Programa Nacional Para a Promoção da Alimentação Saudável, Direção-Geral da Saúde, 1000-123 Lisboa, Portugal; 4 Faculdade de Ciências da Nutrição e Alimentação da Universidade do Porto, 4150-180 Porto, Portugal; 5 EPIUnit—Institute of Public Health, University of Porto, 4050-600 Porto, Portugal; 6 ITR—Laboratory for Integrative and Translational Research in Population Health, 4050-600 Porto, Portugal; 7 Comprehensive Health Research Centre, NOVA Medical School, Universidade NOVA de Lisboa, 1150-082 Lisboa, Portugal

**Keywords:** communication strategies, consumer behavior, consumer segmentation, consumption patterns, data science, decision-making process, intangible culture, marketing intelligence, nutritional education, population health

## Abstract

The Mediterranean Diet (MedDiet) is considered an Intangible Cultural Heritage by UNESCO; it is also the world’s most evidence-based eating pattern for promoting health and longevity. This study aims to investigate consumer segmentation based on consumption patterns and identify barriers to adherence to MedDiet. Data were collected in 2020 by telephonic survey based on PREDIMED, using a quota sampling technique by socio-demographic variables, such as gender, age, and regional representation of the Portuguese population. The final sample was composed of a total of 1000 respondents. The main results show that regardless of the awareness of the MedDiet (62%), Portugal witnessed a loss of the traditional MedDiet, with the exception of the stand-out statistic that 95% of respondents still use of olive oil as the main culinary fat. Five segments were identified: (1) MedDiet lifestyle followers (11%), (2) olive oil lovers (20%), (3) low-sugar diet foods seekers (11%), (4) healthy and balanced diet seekers (28%), and (5) low-fat diet foods seekers (30%). The main barriers to adhering to the MedDiet include lack of knowledge about the MedDiet, education level, financial comfort, and specific food preference, which by segment are: (1) being passionate about soda drinks, (2) an excess of sweets, (3) low level of pasta consumption, and a (5) lack of fruit, vegetables, and legumes. Segment (4) holds a leading position in MedDiet adherence. The main obstacle to consuming fish is its high price, taste, and challenges in cooking it. When it comes to bread and oleaginous nuts, the belief that these foods are “fattening” reduces consumption. Results help to tailor education strategy and increase adherence to the Mediterranean lifestyle.

## 1. Introduction

The scientific community has become increasingly interested in the overall quality of diets rather than in single-nutrient-based approaches to examine diet–health relationships [1,2,3]. At the same time, the traditional Mediterranean Diet (MedDiet) is considered the world’s most evidence-based eating pattern for promoting health and longevity [4,5]. Expert consensus has even identified the MedDiet as the easiest to follow among healthy eating patterns [6]. However, we have witnessed a change in dietary and lifestyle habits that have led to the loss of the traditional MedDiet pattern. Even in Mediterranean countries, traditional eating patterns have progressively given way to alternative habits, with documented decreases in fruit, vegetable, and bean consumption [7]. Factors accounting for this shift include globalization, more poverty and sedentariness, and increased intake of sugars and energy-dense and processed foods [8], which contribute to the current epidemics of obesity [9]. Therefore, with the eventual goal of developing methods to promote and popularize the adoption of evidence-based Mediterranean eating principles, this research was developed.

UNESCO has defined the MedDiet as Intangible Cultural Heritage, i.e., “a set of knowledge passed down from generation to generation, constantly recreated by the communities, with the ability to provide a feeling of identity and continuity while promoting the respect for cultural diversity and human creativity” [10]. The primary features of the Mediterranean dietary pattern were defined in 1993 during the International Conference on MedDiets [11]:

1. A diet characterized by a high intake of plant-based foods, including fruits, vegetables, whole grains, fresh and dried legumes, nuts, and seeds.
2. Preference for fresh, locally sourced, minimally processed, and seasonal food items.
3. Use of olive oil as the primary source of dietary fats.
4. Limited to moderate intake of dairy products, predominantly cheese and yogurt.
5. Rare and minimal consumption of red meat.
6. Regular inclusion of fish in the diet.
7. Moderate to low consumption of wine, primarily during meals.

The traditional MedDiet must adapt to the modern era according to new lifestyles, new agriculture, and environmental constraints [12]. Academic literature has been dedicated to investigating the behavior of consumers of different nationalities concerning the MedDiet, namely their attitudes, drivers, and barriers influencing adherence to the MedDiet, e.g., [13,14,15,16].

Specifically in Portugal, the General Directorate of Health (that in the country of study uses an acronymous of DGS from Direção-Geral da Saúde) is concerned with promoting adherence to the MedDiet as well monitoring adherence to this dietary pattern and evaluating the knowledge of the Portuguese population about it [17,18], recognizing that preserving and promoting this lifestyle is crucial, as it embodies not only a nutritious diet but also the historical, cultural, and habitual practices of the communities that embrace it [19].

Not all consumers are alike, so understanding consumption patterns is crucial for identifying diverse consumer segments and their unique characteristics [19]. This can be achieved through market segmentation, which focuses on consumer motivation and provides insight into their needs [20]. Such understanding allows the development of tailored policies that can increase adherence to the MedDiet [19,21]. There are three perspectives on dietary segmentation—Health and Wellness, Social and Cultural, and Management and Strategy—each including seven approaches, namely Demographic-Based, Lifestyle Segmentation, Health-Driven, Psychographic, Occasion-Based, Geographic and Cultural, and Behavioral Segmentation [3,4,5,6,7,8,9,10,11,12,13,14,15,16,17,18]. Significant differences in culture, ethnic background, religion, and climate contribute to varied dietary practices both between and within countries [19]. Consequently, most studies (excluding those dedicated to medical and agricultural research) focused on segmenting adults regarding the MedDiet are country-specific, including those from Italy [8,14,18,22,23,24,25,26], Cyprus [27], France [22], Greece [14,28], Tunisia and Morocco [14], Ireland [5], Spain [11], Australia [12], the US [20], and Slovenia [14,29]. These studies typically employ methods such as cluster analysis or multinomial logistic regression. Besides the widely recognized importance of segmentation in tailoring education and designing the promotion strategy [18] and the DGS concern, this study marks the first time Portuguese adults have been segmented based on their MedDiet consumption patterns. This focus and the use of multiple data analysis techniques [30] for this purpose highlights the innovative nature of the research. It is important to note that most people react positively to the MedDiet concept and pattern and that a variety of effective methods in a diversity of environments, countries, and cultures for disseminating healthier eating based on MedDiet principles can be implemented [4].

This study aims to investigate consumer segmentation of Portuguese people from the mainland based on MedDiet consumption patterns, to analyze each segment’s demographic variables, and to identify each segment’s barriers to adherence to the MedDiet.

As a result, the specific objectives are as follows:

O1. Identification of consumption patterns.

O2. Identification of different consumer segments based on their consumption patterns.

O3. Analysis of demographic characteristics and the importance of expected benefits from food consumption in each segment.

O4. Identification of the segments that need more attention and the determination of the most important expected benefits in these segments.

MedDiet nutritional education is an education for life and adopting the MedDiet and informing others of its benefits aids in sustaining healthy, good quality lives [31]. The insights provided in this paper can help researchers and practitioners create targeted interventions and health campaigns that are more effective in promoting the MedDiet across different groups.

## 2. Materials and Methods

### 2.1. Procedure and Sample of the Empirical Study

To analyze the evolution of MedDiet consumption patterns and ways to build support for MedDiet promotion, we used DGS data. A survey of the general Portuguese population was conducted to gain an understanding of a general food consumption pattern (see Appendix A).

A total of 1000 respondents participated in the survey. Data were collected in 2020 using quota sampling by using a socio-demographic variable such as sex, age, and regional representation of the population as a non-probability sampling technique. A telephonic medium was the natural option to reach a large population and different ages and geographical regions of the country [32,33,34,35]. Additionally, it ensured a more balanced distribution of power among interview participants, reduced costs and time for large samples, and may have encouraged interviewees to speak freely [35,36,37].

Of the 1000 participants who completed the questionnaire to a standard that was considered valid, 52% were females and 48% were males from the continental part of Portugal, including Alentejo (8%), Algarve (5%), the Center (24%), Lisbon (28%), and the North (35%), with 11% being aged 16–24 years, 12% aged 25–34 years, 13% aged 35–44 years, 15% aged 45–54 years, 23% aged 55–64 years, and 26% aged 65 years or over. As to educational level, 81% were undergraduate respondents and 58% were professionally active (dependent or independent/liberal), which is representative of the population according to the Portuguese Censuses [38]. The financial situation was reported as Tends to be Uncomfortable for 58% and Tends to be Comfortable for 42%. The socio-demographic characteristics of the 1000 participants of this study are shown in Table 1. 

### 2.2. Design and Selection of the Evaluation Instrument

A semi-structured survey was used to collect the data. The scales were developed based on a baseline 14-item PREDIMED questionnaire [39] and was the primary measure used in this study to appraise participants’ adherence to the MedDiet (Appendix A). PREDIMED is known as the largest primary prevention study showing that promoting a Mediterranean diet can reduce the incidence of major chronic diseases in high cardiovascular risk individuals [40,41,42,43,44,45]. In addition, following the suggestion by the DGS, demographic survey questions included MedDiet awareness items, main obstacles to healthy food consumption, and questions that allowed the calculation of Body Mass Index (BMI), food insecurity risk, and financial situation.

The reliability of an assessment tool indicates how consistently and accurately it measures learning. Validity refers to the extent that an assessment tool measures what it is intended to measure [46]. The PREDIMED survey contains 14 questions regarding the consumption frequency of typical and non-typical foods associated with the Mediterranean diet (items listed in Appendix A). Each question is linked to specific characteristics of dietary patterns. In the data analysis, answers that align with these criteria are scored with a “1”. The final score is calculated by summing the individual scores from each question. The total possible score ranges from 0 to 14. Adherence to the Mediterranean dietary pattern is categorized into two levels based on the final score: high (≥10 points) and low (<10 points), according to [39]. As for anthropometric measurements, respondents indicated their weight in kilograms and height in meters, and, subsequently, their Body Mass Index (BMI) was calculated by dividing their weight in kilograms (kg) by the square of their height in meters (m) [40]. Each question was debated by a multidisciplinary team composed of nutritionists, marketing and survey specialists, representatives of commercial companies, statisticians, and people experienced in the nutrition, marketing research, and health sectors. Among them, five professionals hailed from the government and nutrition practitioners, each possessing over 10 years of extensive experience. The remaining five experts were selected from academic backgrounds, each having more than 14 years of research experience relevant to the current study.

Furthermore, the preliminary questionnaire that was established was sent to a group of twenty individuals outside the expert group to assess the clarity, simplicity, and appropriateness of the various questions. During this process, several alterations were introduced, but the overall architecture of the seven sections was kept in the final form of the questionnaire and was anonymous to guarantee a higher level of participation and honesty.

### 2.3. Data Analysis

In this study, data were submitted to different types of analysis. Initially, a descriptive analysis was performed to summarize the sample characteristics. Continuous variables were expressed as means ± standard deviations, while categorical variables were represented as numbers and percentages. Subsequently, factor analysis was primarily used for data reduction, supporting segmentation analyses (clustering) and response modeling, which included multinomial logistic regression and discriminant analysis. Since market segmentation emerged in the late 1950s, the methods have evolved significantly [47]. Segmentation can be approached in various ways, and each method yields a unique segmentation solution. If the objective is marketing communications, factor segmentation might be the approach to use [48]. This method is straightforward to execute, and the results are easy to interpret. Respondents are categorized into segments based on the highest factor score, with each segment reflecting a specific attitudinal or behavioral theme.

The aim of this study is to investigate consumer segmentation based on consumption patterns adapted from the PREDIMED instrument (see Appendix A). Additionally, the analysis considers each segment’s demographic variables and potential barriers. For understanding consumption patterns, Principal Component Analysis (PCA) with Varimax rotation and Kaiser normalization was employed [30,49]. To segment consumers according to these patterns, the K-Means algorithm was utilized [30,50,51,52,53]. Finally, multinomial logistic regression (MLR) was employed to identify the most significant criteria [30,53]—including PREDIMED items, BMI, and socio-demographic data—that influence adherence to the Mediterranean Diet.

- PCA with Varimax rotation and Kaiser normalization is an effective method for analyzing consumption patterns [52,53]. It simplifies complex consumption data by reducing dimensionality, identifies key factors influencing consumption behaviors, and enhances the interpretability of principal components through Varimax rotation. Kaiser normalization further refines the number of components, focusing on the most meaningful variables. This approach enhances reliability and provides actionable insights for targeted decision-making and strategy development, helping to clarify consumer behavior [54].
- The K-Means algorithm is one of the most widely adopted techniques for segmentation in data analysis [52,53]. It is a clustering algorithm that categorizes data into distinct groups, ensuring homogeneity within each cluster. Its value in business lies in its ability to analyze data and uncover insights, helping organizations identify trends, optimize promotional strategies, and make informed decisions. By transforming complex datasets into actionable insights, K-Means supports strategic progress and was chosen here for its simplicity and efficiency [52,53]. This method has been used in several studies to segment individuals based on their adherence to the Mediterranean Diet [26].
- MLR provides several advantages in examining the factors influencing adherence to the Mediterranean Diet [55,56]. It allows for modeling multiple adherence categories, accommodates both continuous and categorical predictors, offers interpretable odds ratios, and provides flexibility to address complex relationships and interactions. These features make MLR an excellent choice for understanding the intricate, multifactorial nature of diet adherence across diverse populations [31].

Quantitative data were analyzed using Statistical Package for Social Sciences (SPSS version 26.0, IBM, Armonk, NY, USA) [51]. Qualitative data were described using the number and percentage. The associations were calculated at a 95% confidence interval and the significance value to measure the strength of evidence was set at *p* < 0.05.

## 3. Results

### 3.1. MedDiet Awareness and Adherence

**Figure 1:**
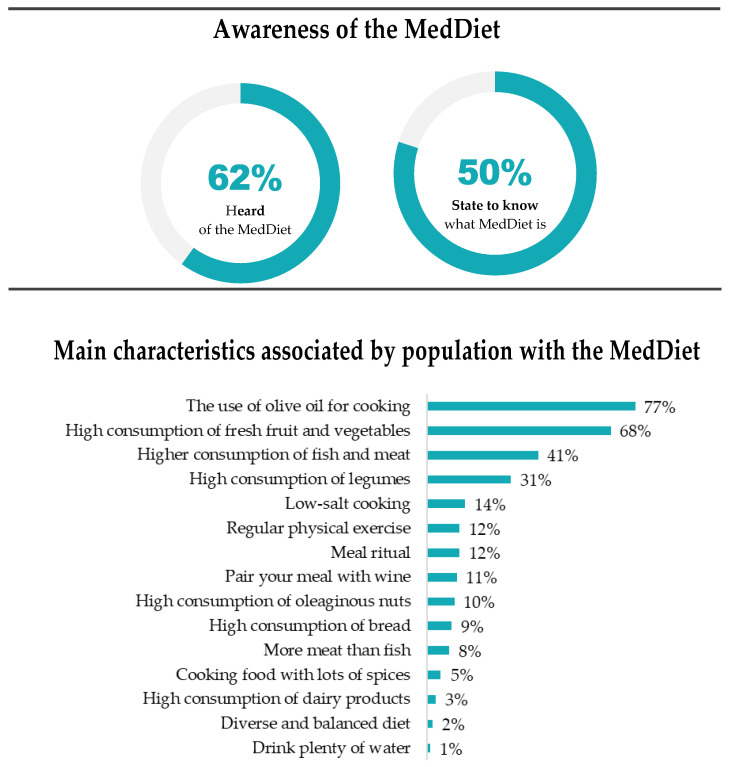
Awareness of the MedDiet in Portugal (Adapted from [17]).

Regarding the awareness of the MedDiet, 62% of respondents say they have heard of MedDiet, and of these, 80% say they know what the MedDiet is (approximately 50% of the total of respondents). When asked about the main characteristics of the MedDiet, “the use of olive oil for cooking” (77%), “high consumption of fresh fruit and vegetables” (68%), and “higher consumption of fish and meat” (41%) were the most frequently mentioned features (Figure 1).

**Figure 2:**
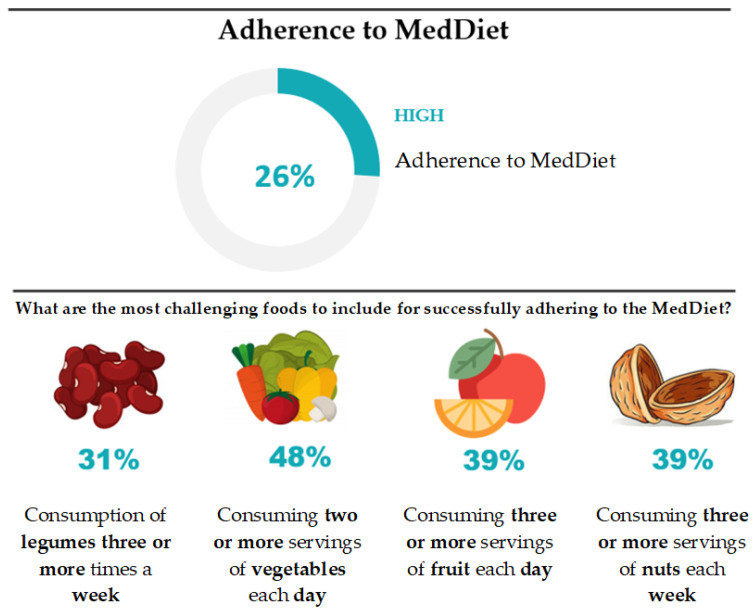
Adherence to the MedDiet in Portugal (Adopted from [17]).

Regarding adherence to the MedDiet, in 2020, 26% of the Portuguese population had a high level, scoring ≥ 10 points on the PREDIMED test. Thus, most Portuguese do not follow this health-protective dietary pattern (<10 points). When analyzing foods where the percentage of inadequate consumption (consumption in quantities less than the recommendations) is higher, legumes (69% with consumption of legumes less than three times a week), vegetables (52% with consumption of fewer than two servings a day), fruit (61% with consumption of fewer than three servings a day) and oleaginous nuts (61% with consumption of fewer than three servings a week), can be highlighted (Figure 2).

Adherence to the Mediterranean food standard has grown 15% since 2016. The high percentage adherence to MedDiet is higher in the group of respondents who know what a MedDiet is (34%).

### 3.2. Anthropometric Measurements

In the questionnaire, the respondents self-administered their weight in kilograms and height in meters, and, subsequently, their Body Mass Index (BMI) was calculated by dividing their weight in kilograms (kg) by the square of their height in meters (m). The BMI results were evaluated according to standards set by the World Health Organization (WHO), where a person is considered overweight if BMI ≥ 25 kg/m^2^ and obese if BMI ≥ 30 kg/m^2^ [57].

The average calculated BMI of females was 24.74 kg/m^2^ ± 4.56, which was significantly lower (*p* = 0.00) than the average BMI of males, which was 26.55 kg/m^2^ ± 4.09. The only significant difference in average BMI was found in age groups older and younger than 44 years (*p* = 0.011), noticing that the 18/24 age group BMI was 22.42 kg/m^2^ ± 3.28, for the 25/34 age group it was 23.49 kg/m^2^ ± 3.98, for the 35/44 age group it was 24.90 kg/m^2^ ± 4.09, for the 45/54 age group it was 25.54 kg/m^2^ ± 3.42, for 55/64 the age group it was 27.73 kg/m^2^ ± 4.59, and for people older than 65 years old it was 26.73 kg/m^2^ ± 4.44. There was a significant difference (*p* = 0.00) between the average BMI of undergraduates (26.10 kg/m^2^ ± 4.47) and graduate respondents (23.54 kg/m^2^ ± 3.64). In addition, the average BMI of respondents with high adherence to the MedDiet was 24.86 kg/m^2^ ± 4.00, which is significantly lower (*p* = 0.001) than all other respondents, 25.88 kg/m^2^ ± 4.55.

### 3.3. MedDiet Consumption Pattern Segmentation

Factor-cluster analysis serves as an alternative segmentation method compared to traditional methods based on consumer demographics [47]. In this study, the authors adopted an approach previously utilized by several other researchers [47,48,49,50]. The factor analysis was used to understand the consumption patterns. Moreover, to segment the consumers according to their consumption patterns, cluster analysis was used, and to determine the most important criteria [40,41] for Mediterranean Diet adherence, MLR was used.

#### 3.3.1. Factor Analysis

In the area of market segmentation, factor analysis typically serves the ancillary role of reducing the many variables available for segmentation to a core set of composite variables (factors) that are used by cluster and regression models [52].

The results of the factor analysis showed high test statistics for Bartlett’s test of sphericity (2893.183, *p* ≤ 0.001) and KMO (0.864), which supports the use of factor analysis for the variables.

Five factor groups, with an Eigenvalue of 1.0 or higher, were the result of the factor analysis. These five factor groups accounted for 61.37% of the total variance. This total variance approached the 60% level suggested by [52,56] for the cumulative percentage of the variance of factorial items. The results shown in Table 2 reveal that all 14 items load significantly on one of the five factor groups.

Five factor groups were retained and assigned the following labels:

- Low-fat diet;
- Balanced diet;
- Low-sugar diet;
- Olive oil dominance;
- Mediterranean style.

#### 3.3.2. Cluster Analysis

We chose the K-Means clustering procedure to specify five clusters. The K-Means algorithm categorizes data for analysis, providing insights for identifying trends, optimizing promotional strategies, and making informed decisions. It simplifies complex datasets to enhance efficiency and support strategic progress [56,58].

In this case, the choice of five clusters is an attempt to see if there are five clusters based on each of the five components we derived from the previous analysis (Table 3). We are looking for clusters of respondents with the same opinions about the overall MedDiet adherence to food habits screeners.

There is a set of characteristics common to all these people. However, there is also a lot that separates them, and there is not a homogeneous group of people but rather five segments with different philosophies of MedDiet consumption patterns.

We can see in the final cluster centers table that the K-Means Cluster 1 (114 respondents), which seems to be characterized by high ratings for Mediterranean style benefits for the diet and the moderate importance of a Low-fat diet, has a negative mean on other factors (Balanced diet, Low-sugar diet, Olive oil dominance). In comparison, Cluster 4 has no negative means on all five factors, especially on a balanced diet. This implies that they rate all aspects of diet as of moderate importance or more. This group is the second biggest segment, with 282 respondents.

Cluster 2 (202 respondents) can be characterized as those consumers who rate Olive oil dominance high but not a Low-fat or Low-sugar diet and Cluster 3 (105 respondents) by Portuguese people who rate a Low-sugar diet high but not a Mediterranean style or Balanced diet.

Cluster 5 is the biggest and represents 297 respondents who follow a Low-fat, but not Balanced diet. Looking across the rows of the Diet component, we can see that there are differences among the five clusters that are large enough to justify our earlier decision to keep it as a component.

Given our ability to see distinct differences among the five clusters based on their attitudes on the five overall benefit issues, we should deem successful both our principal components factoring and our K-Means clustering of the factor scores. Following the analysis of the importance of respondents’ expected benefits in each segment, the analyses of demographic characteristics, behavioral characteristics, and the importance level of benefits are carried out according to diet-expected benefits in each segment and the results are presented in Table 4.

The largest cluster was Segment 5, and the smallest Segments were 1 and 3. In addition, the respondents of Segment 4 are mainly female (63%) with the highest MedDiet adherence point score (81%), and Segment 1 is mainly male (64%) with the lowest MedDiet adherence point score (2%). Segment 4 also has the highest result for the people with normal weight (52%) as well as education level and financial comfort. Segment 3 has the highest level of people with a weight that is more than normal (66% of overweight or obese). Segments 4 and 5 show the biggest success with weight balance with more than half of followers representing Normal level (46% up to normal and 2% under normal weight).

In the following, we will discuss the details of demographic information separately for each section; the most important data regarding each segment are analyzed.

Segment 1. They can be named “MedDiet lifestyle followers” due to the high importance given to products that are widely associated [59,60] with the Mediterranean lifestyle (wine and pasta). This segment includes 11% of respondents, mainly male (64%), aged 55 to 64 years with 12th-year equivalent degree (31%), the highest professional activeness (62%), and a financial situation that tends to be uncomfortable (71%) with the representation of 19% of food insecurity risk (second highest between segments). Even when more than half (53%) of the people in this segment state that they are aware of the MedDiet, only 67% of them (the lowest score between segments) correctly understand it. This group also represents the second-lowest level of people with normal weight (37%) and the highest level of underweight (5%). In terms of specific food consumption, this group is the most passionate about soda drinks (61% do not satisfy Criteria for 1 point) and has the lowest level of nut consumption (only 10% satisfies Criteria for 1 point).

Segment 2. This segment includes “Olive oil lovers”, people who stand out by using olive oil as principal culinary fat more than others. This consumption pattern was more likely to be found in the Center and North of Portugal. This segment includes 20% of respondents, mainly female (57%), older than 35 years. This is the segment with the 2nd highest level of education (22% with complete polytechnic or university courses) and 59% are professionally active (3rd highest) with a financial situation that tends to be uncomfortable for 56%. This segment represents the highest level of food insecurity risk (24%), with only 10% of MedDiet adherence. However, 63% of “Olive oil lovers” are aware of the MedDiet, and 78% of them correctly understand its meaning. This group also represents the second-highest level of people classified as overweight (32%), whilst 46% of “Olive oil lovers” have Normal weight. Regarding specific food consumption, this group is also passionate about sweets (only 51% satisfies the Criteria for 1 point) and nuts (47% satisfy the Criteria for 1 point). Notably, nuts are the least-consumed product among all segments.

Segment 3. This segment includes “Low-sugar diet foods seekers”, the 2nd most preferred by males. The high attention given to the number of soft drinks and commercial sweets consumed per day by the respondents in this segment shows that these people are Low-sugar-diet-oriented. This segment includes 11% of respondents, mainly male (62%), older than 65 years old (44%), and with the lowest level of education (40% do not have more than primary education) and is less professionally active than other segments (47%), with the 2nd most uncomfortable financial situation (62%). However, “Low-sugar diet foods seekers” have the lowest level of food insecurity risk (11%), with only 13% adherence to MedDiet. This is also the segment with the lowest awareness of the MedDiet (48%), noting that 87% of them show a high level of MedDiet understanding. This group also represents the second-highest level of people with weight up to normal (66% of overweight or obese) and the lowest level of those classified as underweight (1%). In terms of specific food consumption, this group also represents the lowest level of pasta consumption (only 14% satisfies the Criteria for 1 point).

Segment 4. “Healthy and balanced diet seekers” are absolute leaders in adherence to the MedDiet (81%) and are highly represented in the North of Portugal (42%), being the 2nd largest segment, which includes 28% of respondents. The high importance of the benefits of vegetables, fruit, legumes, fish, or shellfish consumed per week combined with the quantity of olive oil consumed per day for respondents in this segment (this segment also has the highest MedDiet adherence point score) shows that these Portuguese people are Healthy and balanced diet-oriented and interested in following the MedDiet more consciously (segment with the highest awareness of the MedDiet). This segment includes the highest level of female adherents (63%), mainly up to 55 years old (46%). However, this segment has the largest young group (28% are less than 34 years old) and the highest level of education (a quarter with complete polytechnic or university courses). They are the 2nd most professionally active segment (60%) with the most comfortable financial situation (48%) and 16% of food insecurity risk (24%). Besides the leading position in MedDiet adherence, this group does it most unconsciously, since MedDiet low awareness is 27% in this segment. However, 85% of the people who know it demonstrate a correct understanding. Consequently, more than half of “Healthy and balanced diet seekers” have a normal weight (52%), demonstrating the best BMI.

Segment 5. So-called “Low-fat diet foods seekers” due to the high importance given to low-fat products and the quantity of servings of red meat, butter, margarine, or cream consumed daily, with a preference for consuming chicken, turkey, or rabbit meat. This is the largest segment, which includes 30% of respondents. It is the most balanced segment in terms of gender (53% of female and 47% of male population), mostly presented in the Lisbon region with 30% older than 65 years old. This is the segment with the 2nd lowest level of education (31% with complete primary education or less) and only 58% professionally active (3rd lowest), with a financial situation that tends to be uncomfortable for 57%. This segment represents the lowest level of food insecurity risk (8%) and the 2nd lowest level of MedDiet adherence (5%). However, 39% of “Low-fat diet foods seekers” are aware of the MedDiet, and 78% of them correctly understand its meaning. This group also represents the second-highest level of people with normal weight (half of the segment). In terms of specific food consumption, this group is the one that consumes less fruit, vegetables, and legumes (only 8%, 10%, and 14%, respectively, satisfy Criteria for 1 point) and olive oil (only 13% satisfies Criteria for 1 point). The significate difference (*p* = 0.11) was found in the average calculated BMI of “Healthy and balanced diet seekers” (24.85 kg/m^2^ ± 4.08) compared with “MedDiet lifestyle followers” (26.47 kg/m^2^ ± 5.28) and “Low-sugar diet foods seekers” (26.32 skg/m^2^ ± 4.25).

### 3.4. Barriers to Adherence to the MedDiet

Regarding using olive oil as the main culinary fat, a glass ceiling seems to be reached as it represented 92% of the respondents. The recognition of the DGS communication and education effort is supported by strong brand presence in Portugal [61,62].

Portuguese consumers are an example to the world when it comes to eating fish since Portugal is the EU’s top fish-consuming nation and the country ranks 3rd in the world in that category. This is due to the combination of cultural aspects and government investment. For example, educational campaigns were promoted by Docapesca Portos e Lotas S.A.’s, a government-owned company under the Ministry of Finance and the Ministry of the Sea [20,63].

However, regarding olive oil and fish (21%), the high price is pointed out as the main obstacle to their consumption; also, for fish, the flavor and culinary difficulties also appear as a barrier.

Even when most respondents (53–76%) state that there is no obstacle to consuming foods that predominate in the MedDiet, a relevant percentage (17–31%) of respondents reported already consuming quantities that they consider adequate for these foods. However, for vegetables, soup, and legumes, “not liking the taste” (6%) and the difficulties in cooking (4%) them to obtain tasty meals and appreciation from family members, appear as the main obstacles. For legumes, the perception that these foods “get fat” was also pointed out as an obstacle.

As for bread (30%) and nuts (8%), the perception that these foods “fatten” and that they are even harmful to health (7%) are some of the obstacles to their consumption.

There is still a long way to go to retain more consumers to a healthy diet. It is not an easy task in a world when soda drinks are among the Top 10 most recognizable brands, where no other food brands are present [62]. In this environment, decoding barriers to adherence to the MedDiet becomes fundamental. Using MLR, we examined the association of the MedDiet with different factors, including age, gender, MedDiet awareness, and segment. Binary logistic regression is utilized in those cases when a researcher is modeling a predictive relationship between one or more independent variables and a binary dependent variable (High/Low adherence). Although this is probably the most common form of logistic regression utilized in research literature, other logistic regression models can be useful when your dependent variable is not binary and/or the categories are unordered or ordered [63].

The full model had a significant improvement in fit over a null model (Chi-Square (4) = 70.820 with *p* = 0.00). According to both Deviance and Pearson chi-square tests (*p* = 0.00), the model also exhibits a good fit to the data.

These results contain Likelihood Ratio Tests (Table 5) of the overall contribution of each independent variable to the model. Using the α = 0.05 threshold, we see that all variables were significant. The B column contains regression coefficients (expressed in the metric of log odds). The Exp(B) column contains odds ratios [64]. The fact of knowing MedDiet had the biggest impact on adherence, indicating that it is fundamental to invest in its promotion.

## 4. Discussion

Although the MedDiet has been acknowledged as the best overall diet for the year 2020, it has seen a decrease in its adherence over the past years [65].

**Figure 3:**
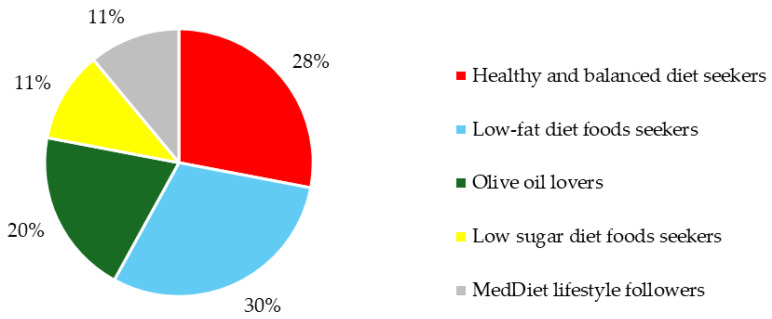
Distribution of the MedDiet segments.

The traditional MedDiet must adapt to the modern era according to new lifestyles, new agriculture, and environmental constraints [12]. In the Portuguese population, one of the main difficulties found in previous studies for MedDiet adherence [15] was the fact this diet is not in line with the fast current lifestyle of modern societies. Moreover, a segmentation in other countries highlighted that both socioeconomic and demographic factors affect the consumption frequency of the main food categories of the MedDiet pyramid [19]. The model presented in this research shows the importance of MedDiet awareness to its adherence. Over the last six years, a world-leading beverage manufacturing company has spent an average of 4 billion dollars a year on advertising worldwide, so investing in a communication strategy has become highly important [66,67]. Consumer segmentation can play an important role in determining a promotion strategy and designing an education campaign; moreover, it can increase its effectiveness and facilitate the innovation process in providing a value-adding strategy [68]. Segmentation makes targeting a particular consumer group easier. In Portugal, five groups were identified (Figure 3) and assigned the following labels according to consumption patterns based on PREDIMED: (1) MedDiet lifestyle followers (11%), (2) Olive oil lovers (20%), (3) Low-sugar diet foods seekers (11%), (4) Healthy and balanced diet seekers (28%), and (5) Low-fat diet foods seekers (30%).

**Figure 4:**
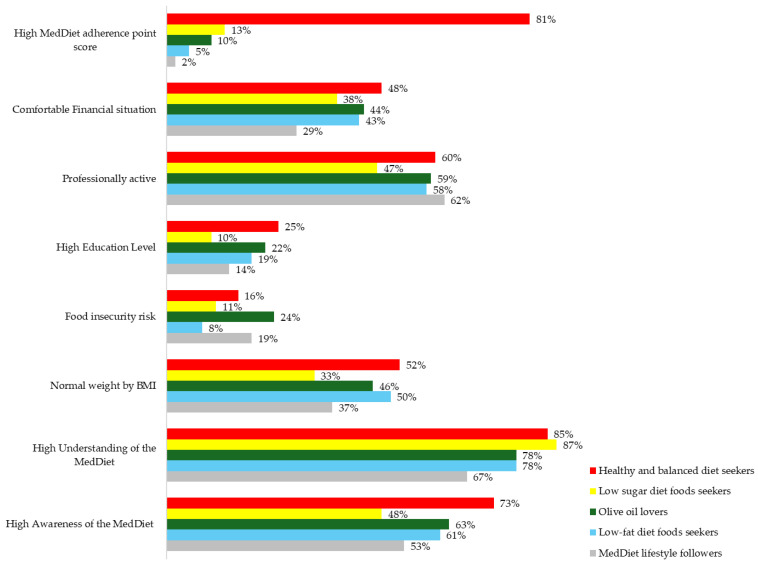
Summary of segmentation patterns for MedDiet consumption. The bar chart shows dietary consumption patterns in Portugal, highlighting key metrics like awareness, understanding, adherence, and socioeconomic factors across various demographics.

As previously mentioned, “Low-fat diet foods seekers” is the largest segment, and “MedDiet lifestyle followers” and “Low-sugar diet foods seekers” are the smallest segments. The “Healthy and balanced diet seekers” segment is mainly female (63%) with the highest MedDiet adherence point score (81%), the “Low-sugar diet foods seekers” segment is the 2nd most preferred by males and the “MedDiet lifestyle followers” segment is mainly male (64%) with the lowest MedDiet adherence point score (2%). The “Olive oil lovers” segment includes 20% of respondents, mainly female (57%) and older than 35 years, and represents the highest level of food insecurity risk (24%), with only 10% of MedDiet adherence. Moreover (Figure 4), the “Low-sugar diet foods seekers” segment has the highest level of people with a weight that is above normal (66% of overweight or obese). The “Healthy and balanced diet seekers” and “Low-fat diet foods seekers” segments show the biggest success with weight balance, with more than half of followers representing Normal level (46% up to normal and 2% under normal weight). This segmentation can help to tailor communication messages. For example, consumers in the “Low-fat diet foods seekers” segment might be targeted with a message such as “MedDiet is a low-fat lunch choice”. In contrast, consumers in the “Healthy and balanced diet seekers” segment might receive messages like “MedDiet will help you maintain”. In this baseline assessment of 1000 participants in the PREDIMED trial, a robust and monotonic inverse association was found between adherence to the MedDiet and indexes of obesity. Our results suggest that closer adherence to an MedDiet is associated with a lower prevalence of obesity. This aligns with conclusions about beneficial metabolic effects in MedDiet studies with rigorously controlled randomized trials [39,69,70,71,72,73,74].

Regarding the awareness of the MedDiet, mainland Portugal is witnessing a loss of the traditional MedDiet lifestyle. Factors accounting for this shift include globalization, more poverty and sedentariness, and the increased intake of sugars and energy-dense and processed foods. Additionally, complexity derives from consumers having individual needs, characteristics, or behaviors. The use of olive oil as the main culinary fat stands out in the big picture and seems to have reached a glass ceiling, representing 92% of the adherents. As an example of the recognition effectiveness of a multi-stakeholder perspective, olive oil use is supported by the DGS´s communication and education efforts and reinforced by strong olive oil product brand presence in Portugal, indicating the need for a different approach to the subject of the MedDiet lifestyle that goes beyond the healthy diet.

The primary lifestyle factors are intrinsic in Mediterranean countries passing from generation to generation and across generations, promoting overall well-being, and transmitting values of hospitality, neighborliness, intercultural dialogue, and creativity [75,76,77]. Urgent action is required from academics and practitioners from a multi-background perspective. This study presents tangible results that can help to support research and the decision-making process of the professionals working in different areas linked with population health, nutritional education, data science, intangible culture, and marketing intelligence. The research carried out by the authors has some limitations, which open opportunities for further research. Repeating this study post-pandemic in the Portuguese context to analyze if there are differences in consumption patterns would be valuable for tracking consumption pattern segmentation. Another opportunity is to extend this study to consumers from other countries, with MedDiet cultures stretching from Italy across Greece, Spain, France, and Turkey to the Middle East [77], to compare segmentation differences in consumption patterns. This could allow a wide comprehensive understanding of the relationship between the Mediterranean lifestyle, consumption pattern, education, and culture [78], considering quality of life [79] and sustainability targets [78,80,81].

## 5. Conclusions

To conclude, the specific objectives of this study were achieved. The identification of consumption patterns of Portuguese mainland consumers and their different segments was obtained based on their consumption patterns. An in-depth analysis was also carried out on the demographic characteristics and importance of the expected benefits of food consumption in each segment to understand the focus of attention for each of them. Finally, examples of communication strategies and actions adapted to each segment were also suggested.

## Figures and Tables

**Table 1:** Sample socio-demographic characteristics.

		Participants	%
Gender	Female	524	52%
	Male	476	48%
	Total	1000	100%
Region	Alentejo	84	8%
	Algarve	51	5%
	Center	237	24%
	Lisbon	280	28%
	North	348	35%
	Total	1000	100%
Age	16/24	112	11%
	25/34	122	12%
	35/44	133	13%
	45/54	151	15%
	55/64	227	23%
	255	255	26%
	Total	1000	100%
Education	Incomplete primary education	55	6%
	Complete primary education	205	21%
	6th year (2nd high school year)	101	10%
	9th year (5th high school year)	212	21%
	12th year (7th high school year/11th year)	234	23%
	Degree/Postgraduate/Masters/Doctorate	193	19%
	Total	1000	100%
Occupational	Looking for work	45	5%
status	Homeowner/domestic	28	3%
	In lay-off	9	1%
	Student (non-worker)	78	8%
	Professionally active (dependent or independent/liberal)	584	58%
	Retired or pensioner	256	26%
	Total	1000	101%
Financial	Tends to be Comfortable	424	42%
situation	Tends to be Uncomfortable	576	58%
	Total	1000	100%

**Table 2:** Information about research’s main factors.

Factors Number	Factor Name	Number of Indicators	Description of Indicators	Factor Load	Cronbach’s Alpha
1	Low-fat diet	3	Quantity of servings of red meat consumed per day (1 serving: 100–150 g)	0.692	0.855
			Quantity of servings of butter, margarine, or cream consumed per day (1 serving: 12 g)	0.633	
			Preference for consuming chicken, turkey, or rabbit meat	0.541	
2	Balanced diet	5	Quantity of vegetable servings consumed per day? (1 serving: 200 g [consider side dishes as half a serving])	0.675	0.846
			Quantity of fruit units (including natural fruit juices) consumed per day	0.573	
			Quantity of servings of legumes per week? (1 serving: 150 g)	0.565	
			The number of servings of fish or shellfish consumed per week (1 serving 100–150 g of fish or 4–5 units or 200 g of shellfish)	0.515	
			Quantity of olive oil consumed per day (including the oil used for frying, salads, out-of-house meals, etc.)	0.318	
3	Low sugar diet	2	Quantity of soft drinks consumed per day	0.451	0.751
			The number of times per week commercial sweets or pastries (not homemade) are consumed.	0.807	
4	Olive oil dominance	1	Use of olive oil as principal culinary fat	0.798	0.748
5	Mediterranean style	3	The number of times per week pasta is consumed	0.714	0.716
			Quantity of wine consumed per week	0.583	
			Quantity of servings of nuts (including peanuts) consumed per week (1 serving 30 g)	−0.332	

**Table 3:** Final cluster centers.

Factors		Segment 1	Segment 2	Segment 3	Segment 4	Segment 5
1	Low-fat diet	0.13102	−1.49937	0.07423	0.48204	**0.40977**
2	Balanced diet	−0.09137	0.08521	−0.14044	**1.03901**	−0.8302
3	Low sugar diet	−0.39076	−0.44119	**0.46369**	0.19699	0.05409
4	Olive oil dominance	−2.16417	**0.23055**	0.00357	0.31321	0.40594
5	Mediterranean style	**0.23747**	−0.03927	−1.66124	0.32664	0.33927
Number of Cases in each Cluster		114	202	105	282	297
Members of each segment		11%	20%	11%	28%	30%

The highest value for each segment is highlighted in bold.

**Table 4:** Characteristics of each segment (numbers in percent).

Demographic Variables		Segment 1	Segment 2	Segment 3	Segment 4	Segment 5
		MedDiet Lifestyle Followers	Olive Oil Lovers	Low Sugar Diet Foods Seekers	Healthy and Balanced Diet Seekers	Low-Fat Diet Foods Seekers
Members of each segment		11%	20%	11%	28%	30%
Gender	Female	36%	57%	38%	63%	53%
	Male	64%	43%	62%	37%	47%
Region	Alentejo	13%	2%	13%	5%	11%
	Algarve	9%	4%	9%	3%	3%
	Center	21%	32%	18%	25%	22%
	Lisbon	28%	23%	29%	25%	34%
	North	29%	38%	32%	42%	30%
Age	16/24	16%	14%	3%	14%	7%
	25/34	8%	13%	5%	14%	14%
	35/44	16%	18%	9%	12%	12%
	45/54	16%	17%	12%	15%	15%
	55/64	22%	19%	27%	26%	23%
	65 or more	21%	20%	44%	20%	30%
Awareness of the MedDiet	Low	47%	37%	52%	27%	39%
	High	53%	63%	48%	73%	61%
Understanding of the MedDiet	Low	33%	22%	13%	15%	22%
	High	67%	78%	87%	85%	78%
Body mass index (BMI)	Underweight	5%	1%	1%	2%	2%
	Normal weight	37%	46%	33%	52%	50%
	Overweight	35%	32%	48%	35%	29%
	Obesity	20%	17%	18%	11%	17%
	NR	3%	3%	1%	0%	2%
MedDiet adherence point score	Low (<10 points)	98%	90%	87%	19%	95%
	High (≥10 points)	2%	10%	13%	81%	5%
Food insecurity risk	No	81%	76%	89%	84%	92%
	Yes	19%	24%	11%	16%	8%
Education	Incomplete primary education	7%	5%	6%	4%	8%
	Complete primary education	17%	12%	34%	21%	23%
	6th year (2nd high school year)	10%	12%	12%	6%	13%
	9th year (5th high school year)	22%	20%	27%	21%	18%
	12th year (7th high school year/11th year)	31%	29%	11%	23%	20%
	Degree/Postgraduate/Masters/Doctorate	14%	22%	10%	25%	19%
Occupational status	Looking for work	6%	10%	2%	6%	3%
	Homeowner/domestic	1%	2%	4%	2%	3%
	In lay-off	2%	2%	2%	0%	0%
	Student (non-worker)	9%	9%	1%	12%	6%
	Professionally active (dependent or independent/liberal)	62%	59%	47%	60%	58%
	Retired or pensioner	21%	19%	46%	20%	30%
Financial situation	Tends to be Comfortable	29%	44%	38%	48%	43%
	Tends to be Uncomfortable	71%	56%	62%	52%	57%

**Table 5:** Likelihood Ratio Tests and parameter estimates for the high adherence to MedDiet.

	Likelihood Ratio Tests						Parameter Estimates to High Adherence to MedDiet							
	Model Fitting Criteria		Likelihood Ratio Tests										95% Confidence Interval for Exp(B)	
Effect	AIC of Reduced Model	BIC of Reduced Model	−2 Log Likelihood of Reduced Model	Chi-Square	df	Sig.	B	Std. Error	Wald	df	Sig.	EXP (B)	Lower Bound	Upper Bound
Intercept	72,619	745,821	71,819	9506	1	0.002	−1.136	0.373	9256	1	0.002			
Age	722,884	742,515	714,884	6201	1	0.013	−0.11	0.044	6207	1	0.013	0.896	0.821	0.977
Gender	731,215	750,846	723,215	14,532	1	0	−0.579	0.153	14,246	1	0	0.56	0.415	0.757
Segment	740,427	760,058	732,427	23,743	1	0	0.272	0.057	22,438	1	0	1.313	1.173	1.469
Awareness	729,595	749,226	721,595	12,912	1	0	0.585	0.166	12,444	1	0	1.796	1.297	2.486

## Data Availability

The original contributions presented in this study are included in the article. Further inquiries can be directed to the corresponding authors.

## Appendix A

Description of the PREDIMED instrument (14-item questionnaire of MedDiet adherence).

**Questions**	**Criteria for 1 Point**
1. Do you use olive oil as the main culinary fat?	Yes
2. How much olive oil do you consume in a given day (including the oil used for frying, salads, out-of-house meals, etc.)?	≥4 tbsp
3. How many vegetable servings do you consume per day? (1 serving: 200 g [consider side dishes as half a serving])	≥2 (≥1 portion raw or as a salad)
4. How many fruit units (including natural fruit juices) do you consume per day?	≥3
5. How many servings of red meat, hamburger, or meat products (ham, sausage, etc.) do you consume per day? (1 serving: 100–150 g)	<1
6. How many servings of butter, margarine, or cream do you consume per day? (1 serving: 12 g)	<1
7. How many sweet or carbonated beverages do you drink per day?	<1
8. How much wine do you drink per week?	≥7 glasses
9. How many servings of legumes do you consume per week? (1 serving: 150 g)	≥3
10. How many servings of fish or shellfish do you consume per week? (1 serving 100–150 g of fish or 4–5 units or 200 g of shellfish)	≥3
11. How many times per week do you consume commercial sweets or pastries (not homemade), such as cakes, cookies, biscuits?	<3
12. How many servings of nuts (including peanuts) do you consume per week? (1 serving 30 g)	≥3
13. Do you preferentially consume chicken, turkey, or rabbit meat instead of veal, pork, hamburger, or sausage?	Yes
14. How many times per week do you consume vegetables, pasta, rice, or other dishes seasoned with sofrito (sauce made with tomato and onion, leek, or garlic and simmered with olive oil)?	≥2
